# D^R^e^A^mocracy: A Method to Capitalise on Prior Drug Discovery Efforts to Highlight Candidate Drugs for Repurposing

**DOI:** 10.3390/ijms25105319

**Published:** 2024-05-13

**Authors:** Kyriaki Savva, Margarita Zachariou, Marilena M. Bourdakou, Nikolas Dietis, George M. Spyrou

**Affiliations:** 1 Bioinformatics Department, The Cyprus Institute of Neurology and Genetics, Nicosia 2370, Cyprus; kyriakis@cing.ac.cy (K.S.); margaritaz@cing.ac.cy (M.Z.); marilenab@cing.ac.cy (M.M.B.); 2 Experimental Pharmacology Laboratory, Medical School, University of Cyprus, Nicosia 2115, Cyprus; dietis.nikolas@ucy.ac.cy

**Keywords:** drug repurposing, clinical trials, neurodegenerative diseases, weight-modulated majority voting

## Abstract

In the area of drug research, several computational drug repurposing studies have highlighted candidate repurposed drugs, as well as clinical trial studies that have tested/are testing drugs in different phases. To the best of our knowledge, the aggregation of the proposed lists of drugs by previous studies has not been extensively exploited towards generating a dynamic reference matrix with enhanced resolution. To fill this knowledge gap, we performed weight-modulated majority voting of the modes of action, initial indications and targeted pathways of the drugs in a well-known repository, namely the Drug Repurposing Hub. Our method, D^R^e^A^mocracy, exploits this pile of information and creates frequency tables and, finally, a disease suitability score for each drug from the selected library. As a testbed, we applied this method to a group of neurodegenerative diseases (Alzheimer’s, Parkinson’s, Huntington’s disease and Multiple Sclerosis). A super-reference table with drug suitability scores has been created for all four neurodegenerative diseases and can be queried for any drug candidate against them. Top-scored drugs for Alzheimer’s Disease include agomelatine, mirtazapine and vortioxetine; for Parkinson’s Disease, they include apomorphine, pramipexole and lisuride; for Huntington’s, they include chlorpromazine, fluphenazine and perphenazine; and for Multiple Sclerosis, they include zonisamide, disopyramide and priralfimide. Overall, D^R^e^A^mocracy is a methodology that focuses on leveraging the existing drug-related experimental and/or computational knowledge rather than a predictive model for drug repurposing, offering a quantified aggregation of existing drug discovery results to (1) reveal trends in selected tracks of drug discovery research with increased resolution that includes modes of action, targeted pathways and initial indications for the investigated drugs and (2) score new candidate drugs for repurposing against a selected disease.

## 1. Introduction

“…And if you find her poor, Ithaka won’t have fooled you. / Wise as you will have become, so full of experience, / you’ll have understood by then what these Ithakas mean.” (C. P. Cavafy, “Ithaka” from C. P. Cavafy: Collected Poems. Translation copyright © 1975, 1992 by Edmund Keeley and Philip Sherrard. Princeton University Press, 1975.) These are the last three verses of the poem “Ithaka” from the Greek poet Constantine Cavafy that deals with the value of the journey in relation to the value of the destination. This is more relevant than ever in the case of the countless journeys to discover new drugs where the destination is often not what we have expected. Although the results of most of the drug discovery trajectories are not successful, the question remains whether we can integrate this knowledge and experience in a democratic way into computational methods that will increase the chances for the next drug discovery efforts to deliver success. 

According to the literature, only a few studies have used this massive amount of knowledge to gain further insight into the drug discovery process. Himmelstein et al. constructed a network that integrated knowledge from a very large number of biomedical studies [1]. Data were integrated from 29 public resources to connect compounds, diseases, genes, pathways, side effects, symptoms and other information. This approach described more than two million relationships among data points, which could be used to develop models that predict which drugs currently used in the clinic might be best suited to treating any of the 136 common diseases. Moreover, Kropiwnicki et al. created a tool, named DrugShot (https://maayanlab.cloud/drugshot/, accessed on 29 April 2024), that prioritises drugs and small molecules associated with biomedical search terms. Apart from listing known associations, DrugShot predicts additional drugs and small molecules related to any search term [2]. In another study, Zhu et al. integrated multiple drug-knowledge databases to develop a drug-knowledge graph to predict drug repurposing candidates by using machine learning models [3].

However, to the best of our knowledge, there have been no studies to date that have collectively capitalised on the already available information that has been generated in both computational drug repurposing studies and clinical trials with increased resolution, including modes of action, targeted pathways and initial indications for the investigated drugs.

Drug repurposing or repositioning, which describes the identification of novel uses for existing drugs, has attracted considerable attention during the past few decades, as it offers a cost-efficient and time-effective alternative avenue to therapeutics compared to de novo drug discovery [4,5,6]. In general, traditional drug repurposing attempts to reveal the effect of a drug and its mechanism of action [7], screens available drugs against new targets to reveal novel drug indications [8], investigates drug characteristics such as their structures and side effects [9] or explores the relationships of drugs with diseases [10]. Up to now, several in silico drug repurposing efforts have been developed including network-based approaches, transcriptomic approaches, structure-based and ligand-based approaches, machine learning approaches, etc. [11,12,13,14].

So far, there have been many studies on computational drug repurposing that have attempted to highlight candidate repurposed drugs, as well as clinical trial studies that test drugs in different phases. Both approaches result in long lists of drugs that are usually not exploited further. To the best of our knowledge, this information has not been extensively exploited collectively. We posed the following questions: (1) How can we integrate the available information? (2) How can we leverage the integrated knowledge to generate a knowledge-based model to assist drug discovery?

This available a priori knowledge can turn into novel information regarding a specific drug of interest. To do so, we designed and developed a methodology named D^R^e^A^mocracy, which exploits this huge pile of information, in a resolution beyond the nominal reference of the drug per study. Specifically, information such as the drugs’ mechanisms of action (MoAs) with their initial indications (Inds), as retrieved from the Drug Repurposing Hub, and the targeted pathways (Paths), as obtained from the KEGG Pathway Database, is subjected to weight-modulated majority voting schemes to score and finally prioritise the candidate drugs against a disease. This methodology is not a predictive model; yet, it gives scoring power to the user based on user-selected preferences, offering a quantified aggregation of existing drug discovery results.

D^R^e^A^mocracy was applied to four diseases of the neurodegeneration spectrum as a testbed to check the usefulness of this method. Neurodegenerative diseases are a group of diseases that present many challenges in terms of the effective availability of treatments, making them a great candidate for drug repurposing. For this reason, we chose two well-studied diseases, Alzheimer’s Disease (AD) and Parkinson’s Disease (PD), for which there is a lot of information available on repurposing studies and clinical trials, and two diseases with less available information, Multiple Sclerosis (MS) and Huntington’s Disease (HD).

## 2. Results

### 2.1. Reference Tables for Each Disease, Type of Analysis and Drug Feature

As previously discussed, lists of candidate repurposed drugs from computational drug repurposing studies, as well as clinical trial studies for four neurodegenerative diseases (AD, PD, HD, MS), were collected to utilise this integrated information available for the better and more effective suggestion of repurposed drugs for a given disease.

**Figure 1:**
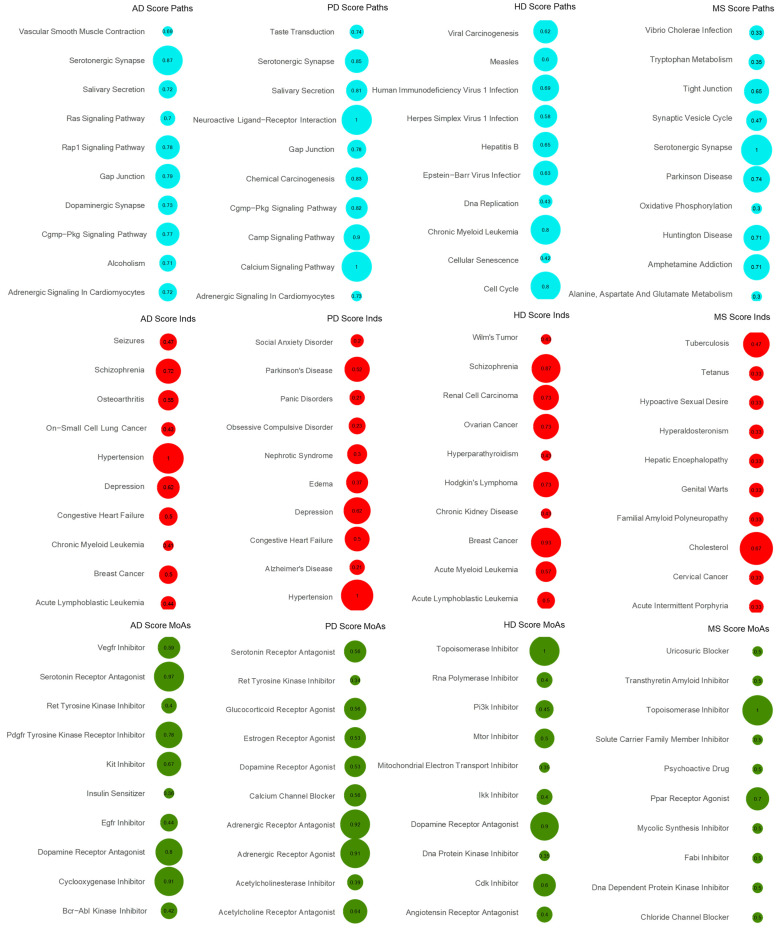
DC-matrix for CDRS. DC-matrices were generated for each disease per signature (Paths, MoAs and Inds) using the CDRS lists. The top 10 scored features are shown for each disease in a bubble grid chart. The number in the bubbles indicates the DC score of each modality (also encoded as the bubble size). Blue colour depicts Paths, red colour depicts initial Inds and green colour depicts MoAs.

By calculating the DC score for the aggregated MoAs, Paths and initial Inds, we detected the top-ranking ones per disease and drug collection method. The DC-matrix for the AD CDRS list revealed as top scoring drug features the serotonergic synapse, serotonin receptor antagonism and hypertension for Paths, MoAs and initial Inds, respectively. Serotonin receptor antagonism was also found in the top four MoAs for PD CDRS and CTS DC-matrices, with a DC score of 0.56 and 0.75, respectively. For the initial Inds, hypertension was detected as not only the highest scoring one for AD but also for the PD CDRS DC-matrix table (Figure 1). There are many studies and meta-analyses available that show the association between antihypertensives and AD and specifically the effect that they have in decreasing the risk of AD [15,16,17]. Moreover, antihypertensives, such as centrally acting dihydropyridine and high cumulative doses of ACEIs, were shown to have a possible association with reduced PD incidence in hypertensive PD patients [18].

The cAMP signalling pathway, which was identified as the highest scoring pathway in HD CTS DC-matrix tables (with a score of 1) and in the top three of PD CDRS and CTS (0.9 and 0.79, respectively), has been shown to play an important role in mediating neurotransmitters and regulating numerous cell functions, such as synaptic plasticity in neurons. In PD, the dysregulated cAMP signalling pathway is associated with levodopa-induced dyskinesia, whereas its dysfunction is also found in HD [19,20].

**Figure 2:**
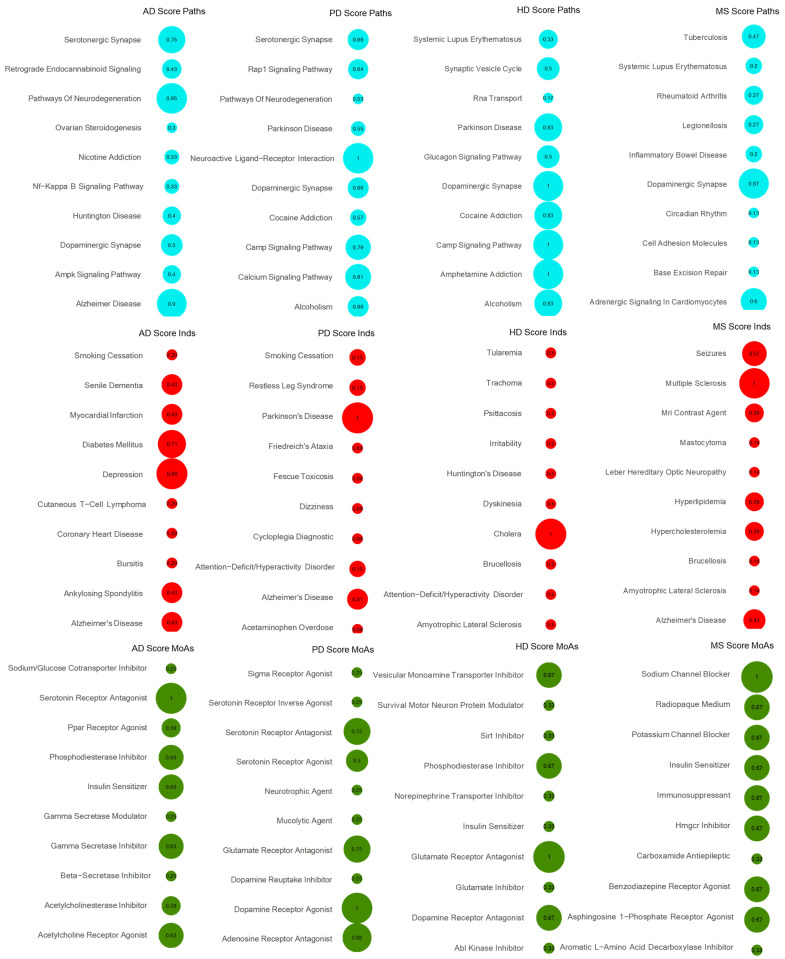
DC-matrix for CTS. DC-matrices were generated for each disease per signature (Paths, MoAs and Inds) using the CTS lists. The top 10 scored modalities are shown for each disease in a bubble grid chart. The number in the bubbles indicates the DC score of each modality (also encoded as the bubble size). Blue colour depicts Paths, red colour depicts initial Inds and green colour depicts MoAs.

Moreover, topoisomerase inhibitor was detected as the highest scoring MoA in both the HD and MS CDRS reference tables, with a score of 1 in both diseases. The DNA topoisomerase inhibitor mitoxantrone is a drug used for patients with worsening relapsing–remitting and secondary progressive Multiple sclerosis and hepatocellular carcinoma [21,22]. On the other hand, in the CTS DC-matrix, glutamate receptor antagonist and sodium channel blocker were detected as the top ones, respectively (Figure 2). As shown by Abd-Elrahman et al. (2017), blocking the metabotropic glutamate receptor in an HD mouse model showed an improvement in cellular, motor, and cognitive skills [23]. In addition, the sodium channel blocker safinamide, which was tested in phase III trials for PD, could also be used for MS to protect against axonal degeneration according to Morsali and colleagues, as it preserved the integrity and function of the axon in two rat models of MS [24]. Full lists of DC-matrices for CDRS and CTS per disease are available in Supplementary Tables S1–S8.

### 2.2. Common Signatures in CTS vs. CDRS for Neurodegenerative Diseases

**Figure 3:**
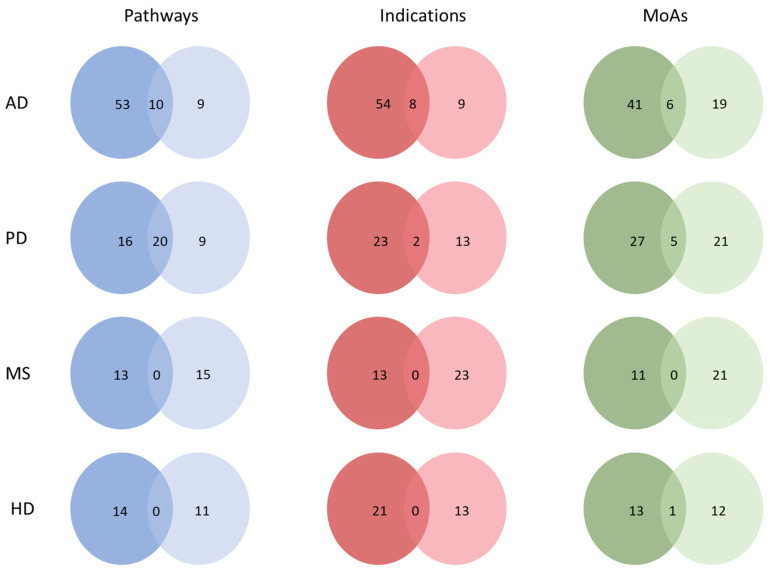
Signature comparison across different diseases. CDRS represents signatures detected from drugs in computational drug repurposing lists and CTS represents signatures detected from drugs in current clinical trials for each disease, respectively. CDRS lists are depicted using a darker colour and CTS lists are depicted with a lighter colour for each property of the drugs (Pathways, Indications and MoAs).

As a next step, we compared the CDRS and CTS signatures (MoAs, Inds and Paths) detected for each disease independently. The top signatures were selected using a hypergeometric test and a *p*-value of <0.05, as shown in Figure 3. For AD, commonalities in CDRS and CTS were found across all components of the signature. For MoAs, six commonalities were found, some of which are highly associated with AD. These include the serotonin receptor antagonist, acetylcholinesterase inhibitor and HMGCR inhibitors. All three MoAs have been associated with neurodegeneration, specifically AD [25,26,27]. Moreover, at present, acetylcholinesterase inhibitors, such as donepezil and rivastigmine, are the main class of drugs used for the treatment of AD [27].

For initial Inds, eight commonalities were detected. These include cutaneous T-cell lymphoma (CTCL), depression, heart attack, ankylosing spondylitis, bursitis, coronary heart disease, exercise-induced bronchoconstriction (EIB) and myocardial infarction. HDAC inhibitors such as romidepsin and vorinostat, used for the treatment of CTCL, have been reported to exhibit neuroprotective actions, either through the suppression of pro-apoptotic factors or through the release of pro-inflammatory factors of activated microglia [28,29]. Furthermore, a history of depression may increase the risk of developing AD later in life [30]. Additionally, ten common Paths were found: serotonergic synapse, the AMPK signalling pathway, dopaminergic synapse, viral myocarditis, ovarian steroidogenesis, glycerophospholipid metabolism, the PPAR signalling pathway, circadian rhythm, arachidonic acid metabolism and notch signalling pathway.

For PD, commonalities in CDRS and CTS were found across all components of the signature, as shown for AD. For MoAs, five commonalities were detected such as dopamine receptor agonist, serotonin receptor antagonist, dopamine precursor, aromatic L-amino acid decarboxylase inhibitor and norepinephrine precursor. Several dopamine receptor agonists have been approved by the FDA for PD [31].

For the initial Inds of PD, two commonalities were found between CDRS and CTS of PD and AD. Furthermore, 20 common Paths were identified, such as the dopaminergic synapse, serotonergic synapse, cAMP signalling pathway, neuroactive ligand–receptor interaction, calcium signalling pathway and others, some of which will be discussed in the next sections.

For HD, commonalities in CDRS and CTS were found only in MoAs. Specifically, one common element was detected, the dopamine receptor antagonist. One of the two FDA-approved drugs for HD, known as tetrabenazine, depletes dopamine by inhibiting the vesicular monoamine transporter 2 (VMAT2) [32]. On the other hand, for MS, no commonality was detected in either signature. These commonalities across CDRS and CTS suggest that repurposing studies and clinical trials are partially in agreement; however, there are also many unique findings. A hypergeometric test was conducted between the CDRS and CTS for the different diseases and modalities in order to assess the significance of overlap between the two lists. Statistically significant differences were detected for AD and PD across all modalities and for HD, only for MoAs (*p*-value < 0.05). Detailed *p*-values for all comparisons can be found in Supplementary Table S9.

We have also performed linear regression analysis to test the fit of frequencies between CDRS and CTS, and we can see that the ranking of values is consistent (Supplementary Table S10 and Figure S1).

### 2.3. Commonalities and Differences in Signatures across Neurodegenerative Diseases

For the next part of the analysis, we wanted to find commonalities across the four neurodegenerative diseases under investigation to detect common patterns that could possibly be observed behind neurodegeneration.

**Figure 4:**
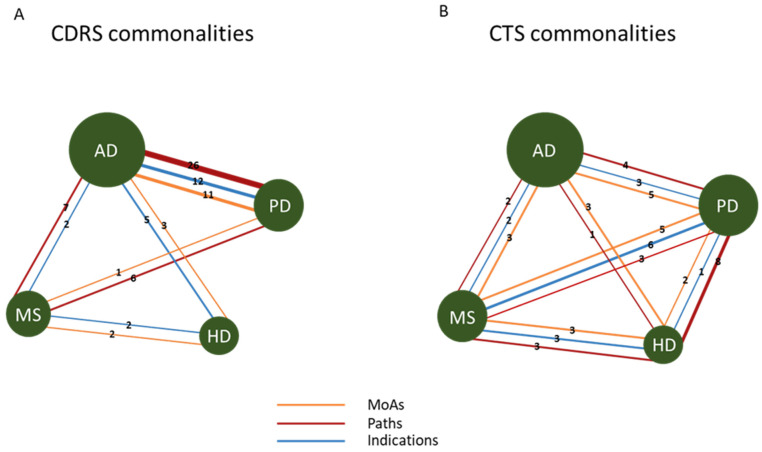
Commonalities across the four neurodegenerative diseases. Disease–disease network. Nodes are the four neurodegenerative diseases, and edges are the MoAs, Paths and Inds. (**A**). Network of CDRS commonalities. (**B**). Network of commonalities of CTS. Edges indicate the number of MoAs, Paths and Inds that are shared between diseases. The size of the nodes indicates the number of drugs available for each disease. Red colour edges indicate the Paths, orange colour indicates the MoAs and blue colour indicates indications.

A disease–disease network was created to show commonalities between CTS and CDRS for all diseases (Figure 4). In CTS, results are more consistent and all components of the signature (MoAs, Paths and Inds) are shared by all four neurodegenerative diseases. On the other hand, for CDRS lists, for the two diseases (AD and PD) for which data were more abundant, we observed a stronger consistency in all components of the signature. Connections with MS and HD are weaker, with AD and HD sharing only Inds, HD and MS sharing MoAs and Inds and MS and PD sharing Paths and MoAs, whereas PD and HD have zero commonalities.

More specifically, for disease comparisons for initial Inds using the CDRS lists, commonalities were only found pairwise. For example, for AD and HD, acute lymphoblastic leukaemia (ALL) and schizophrenia were detected, among others. One of the drugs that is used to treat leukaemia in adults is amsacrine, a topoisomerase inhibitor. As previously discussed, mitoxantrone, which is also a topoisomerase inhibitor, is used for treating worsening relapsing–remitting and secondary progressive MS [33]. Hence, a connection between ALL and neurodegenerative diseases could be indicated.

For AD and PD, depression, hypertension and others were detected. For MoAs, the serotonin reuptake inhibitor was detected as the common MoA among AD, PD and MS. Drugs in this category are used in neurology/psychiatry, such as the anti-depressants paroxetine, trazodone and others. For AD and HD, angiotensin receptor antagonist and dopamine receptor antagonist were detected. For AD and PD, 10 common MoAs, such as acetylcholinesterase inhibitor and serotonin receptor antagonist, were identified. Inhibitors of the acetylcholinesterase enzyme, which breaks down the neurotransmitter acetylcholine, are frequently used for the treatment of neurodegenerative diseases [31].

For Paths, amphetamine addiction, serotonergic synapse and others were detected for AD, PD and MS. For AD and MS, ABC transporters and tight junction pathways were detected. In addition, for PD and MS, phenylalanine metabolism was detected. The ABC transporters pathway is targeted by diabetes mellitus and hyperglycaemia drugs, such as repaglinide. There is evidence to support the idea that people with type II diabetes are at increased risk of developing all types of dementia [34]. For AD and PD, a total of 22 Paths were detected; AMPK signalling pathway, calcium signalling pathway and dopaminergic synapse were detected, among others. AMPK activation has been shown to play a preventive role in AD, as many studies have shown [35]. On the other hand, other studies have reported that AMPK activation has an aggravating effect on the development of AD [36]. These findings make the therapeutic potential of AMPK in AD controversial.

For disease comparisons for Inds using the CTS lists, Alzheimer’s disease was found to be a common indication for all four neurodegenerative diseases. In addition, mastocytoma was found to be common in AD and MS. Friedreich’s ataxia, among others, was found to be common in PD and MS. Senile dementia was found to be common in AD and PD, whereas Amyotrophic Lateral Sclerosis (ALS) and hypercholesterolemia were found to be common in MS and HD, among others. The norepinephrine transporter inhibitor was found to be a common MoA among the four neurodegenerative diseases. Changes in norepinephrine reuptake, which are carried out by the norepinephrine transporter, are observed in many neurodegenerative diseases, such as AD and PD [37,38].

Moreover, glutamate release inhibitor and serotonin receptor antagonist were found to be common in AD and PD, among others. The glutamate inhibitor was found to be common in HD and MS, while the calcium channel modulator was found to be common in PD and MS, among others. Alterations in calcium channels have been implicated in several neurodegenerative diseases, such as AD, PD and HD [39]. For disease comparisons for Paths, dopaminergic synapse was found to be a common indication in all four neurodegenerative diseases. Alterations in the dopaminergic system are more common in PD, where a large proportion of dopaminergic neurons in the Substantia Nigra pars compacta are lost, but are also frequent in AD [40,41]. The complete lists of comparisons can be found in Supplementary Tables S11 and S12.

### 2.4. Generation of the Super-Reference Table of Drugs

Once the individual tables for each signature were created, the next step was to generate a single reference table for each disease, including a Total Composite Score for all components of the signature (MoAs, Paths and Inds) for both collection methods. A snapshot of the super-reference table generated using the drug library of the drug repurposing hub is shown in Table 1 (Supplementary Table S13). The 100 highest-scoring drugs for each disease were selected using the Total Composite Score.

**Figure 5:**
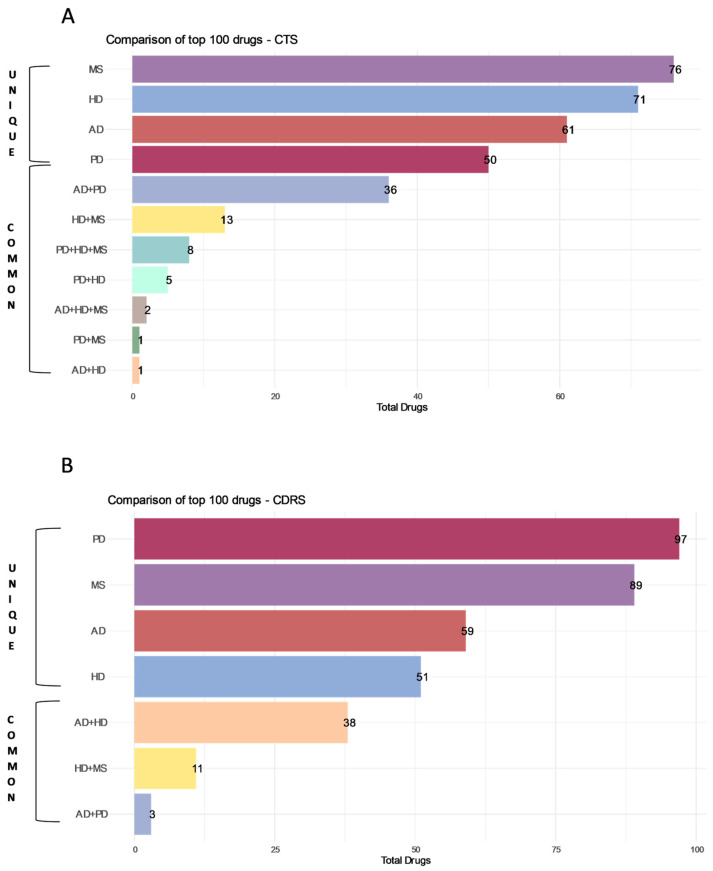
Comparison of the top 100 drugs generated from the super-reference table: (**A**). Comparison of top 100 drugs using CTS and (**B**) Comparison of top 100 drugs using CDRS. The top 100 drugs generated from the super-reference table of the Drug Repurposing Hub for AD, PD, HD and MS were compared. Unique drugs for each disease are shown, as well as commonalities among diseases.

From the CDRS Composite scores, common drugs were detected between pairs of diseases. For instance, three common drugs were detected between AD and PD (ketanserin, agomelatine and mirtazapine). For AD and HD, 38 common drugs were found, such as iloperidone, olanzapine, amisulpride and risperidone. For HD and MS, 11 common drugs were found in the top 100 drugs for both diseases, including idarubicin, daunorubicin, amsacrine and teniposide (Figure 5B).

Regarding the CTS Total Composite Scores, common drugs were detected between two diseases, as observed in the CDRS lists; yet, commonalities were also detected among three diseases. Two common drugs were detected in AD, HD and MS (pipotiazine–palmitate, methylergometrine), while eight common drugs in PD, HD and MS (such as gyki-52466, ly215490, sym-2206 and talampanel). Additionally, one common drug was detected in AD and HD, as well as in PD and MS (clozapine and lamotrigine, respectively). Moreover, five common drugs were found in PD and HD such as budipine, amantadine and topiramate. Lastly, the highest number of commonalities was detected between AD and PD, with 36 common drugs, such as metergoline, tropisetron, zacopride and methysergide, detected (Figure 5A).

### 2.5. Scoring New Candidate Drugs for Repurposing against a Selected Disease

To present an application of the scoring part of D^R^e^A^mocracy, we scored different groups of drugs that are related to AD, such as the current FDA-approved drugs, drugs that previously failed in clinical trials for AD, and three different groups of candidate repurposed drugs that we suggested in our previous work for stage-specific drug repurposing for AD and for Braak stages I–II, III–IV and V–VI [42] (which were intentionally omitted from the sources used to compile the CDRS reference table). 

**Figure 6:**
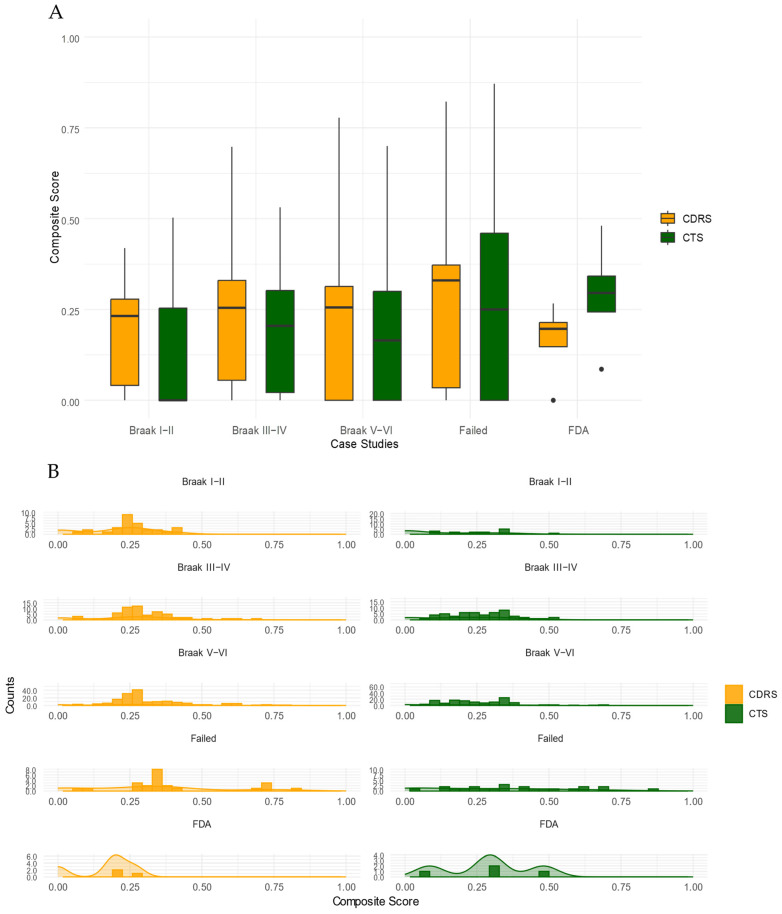
Plots of Composite Scores for AD case examples. (**A**). Box plots of Composite Scores of selected AD-related drugs (Braak I–II, Braak III–IV, Braak V–VI, FDA-approved and Failed) were generated using the CDRS and CTS reference tables. Black dots show the outliers. (**B**). Distribution histogram of Composite Scores of selected AD-related drugs using the CDRS and CTS reference tables.

Based on the output of D^R^e^A^mocracy, we compared the Composite Scores of the different drug groups that were tested. Figure 6 shows the comparison of the different drug groups, using the Composite Scores of selected AD-related drugs (Braak I–II, Braak III–IV, Braak V–VI, FDA approved and Failed drugs) of the CDRS and CTS reference tables, as illustrated with boxplots (Panel A) and distribution histograms (Panel B). The Wilcoxon test was carried out to test whether the D^R^e^A^mocracy scores are significantly different across the types of scores and groups of drugs of interest. Paired comparison of Braak I–II data using CDRS and CTS shows statistical significance with a *p*-val = 0.004. Moreover, paired comparisons of Braak III-IV and Braak V–VI data using CTS and CDRS data show statistical significance with a *p*-val = 0.026 and 1.21 × 10^9^, respectively. When comparing the groups between them, statistical significance was found only for the comparison of Braak I–II with Braak III–IV for the CTS reference table, with a *p*-val = 0.009.

The output of D^R^e^A^mocracy in these test drug queries gives a snapshot of the trends regarding the selected drug categories and the existing knowledge from CTS and CDRS; FDA-approved drugs present higher Composite Scores, as expected, in CTS than in CDRS. CTS are expected to include candidate drugs with more similarity to the already FDA-approved drugs, whereas the candidate drugs from CDRS are expected to present higher diversity from the already approved drugs.

Additionally, failed drugs for AD have a higher median of Composite Score values in CDRS than in CTS. Failed drugs could be supported more by the a priori knowledge from CDRS than by the a priori knowledge from CTS, as expected, since the diversity provided by the CDRS is higher than the one by CTS.

Among the three stage-specific groups of candidate repurposed drugs from our previous study [42], the candidate drugs for Braak stage I–II (incipient AD stage) are shown to be more unexpected based on the a priori knowledge from both the CDRS and CTS collection methods. There is a noteworthy difference in the median values of the Scores between the two collection methods in favour of CDRS, as expected. Candidate drugs for Braak stages III–IV (moderate AD stage) and V–VI (severe AD stage) also present higher Composite Scores in CDRS than in CTS, suggesting that our computational repurposing study follows the trend of the a priori knowledge of the rest of the CDRS, as expected.

## 3. Discussion

In this work, we proposed a methodology, D^R^e^A^mocracy, aiming to utilise a priori knowledge to reveal trends in selected tracks of drug discovery research with an increased resolution that includes MoAs, Paths and Inds for the investigated drugs, as well as to score new candidate drugs for repurposing against a selected disease. This methodology is not designed to be a predictive model but instead offers a quantified aggregation of existing drug discovery results based on user-defined preferences. Four neurodegenerative diseases have been used as a testbed for the application of this methodology. Our analysis generated frequency tables, known as DC-matrices, for each disease based on the MoAs, Paths and Inds of the drugs detected from the CDRS and CTS. Based on these frequency tables, we constructed a super-reference table with drug suitability scores for each of the four neurodegenerative diseases. Hence, a given set of drugs can be scored based on the super-reference table concerning its proposal in other studies. Moreover, disease–disease commonalities regarding Paths, MoAs and Inds were detected to highlight common mechanisms of neurodegeneration.

Upon the detection of the top candidate repurposed drugs, an in-depth investigation can take place using diverse tools and databases. Notably, ChEMBL (https://www.ebi.ac.uk/chembl/, accessed on 29 April 2024) emerges as a pivotal resource, offering critical insights into the pharmacokinetics of the drugs of interest and their targets, such as IC50 and Ki values. Through ChEMBL, users can search for a drug of interest and access detailed drug profiles, including drug classification, structural representation (SMILES, Molfiles, InChi Keys), drug indications, drug mechanisms of action (highlighting targets and action types), structurally similar drugs and other pharmacokinetic parameters. These pharmacokinetic parameters include the standard value for IC50 or Ki, the assay description used, the target name and type, etc. Additionally, BindingDB (https://www.bindingdb.org/rwd/bind/index.jsp, accessed on 29 April 2024) is another resource providing insights into target interactions (e.g., Ki, IC50, Kd) and compound properties (e.g., chemical structures, SMILES notation). Also, the Therapeutic Target Database (TTD) (https://idrblab.net/ttd/, accessed on 29 April 2024) is another database that provides essential information on the drugs and their therapeutic targets, focusing on the associated pathways of the corresponding drugs. The AdmetSAR web application (http://lmmd.ecust.edu.cn/admetsar2/, accessed on 29 April 2024) and SwissADME (http://www.swissadme.ch/index.php, accessed on 29 April 2024) can be used to facilitate in silico assessment of the absorption, distribution, metabolism, excretion and toxicity (ADMET) properties of compounds, enabling the prediction of pharmacokinetic properties and drug-likeness essential for drug discovery efforts. Moreover, the SIDER database (http://sideeffects.embl.de/, accessed on 29 April 2024) can serve as a key resource for investigating the side effects of associated marketed medicines. SIDER provides insights into side effect frequencies, drug classifications and links to detailed drug–target relationships.

As revealed using our methodology, MoAs such as serotonin receptor antagonism and serotonin reuptake inhibitor were found to be highly scored in AD, PD and MS. Serotonin receptors are abundant in the central nervous system and specifically in cognitive and learning brain regions. Common anti-depressants that are used in many neurodegenerative diseases target specific serotonin receptors. Apart from the anti-depressive effect they have, there have been pre-clinical studies that showed a strong impact of anti-depressants on microglial cells, whose activation plays an essential role in the pathogenesis of several neurodegenerative diseases. Investigating the effects of anti-depressants on microglial cells showed a significant decrease in microglial activation markers after anti-depressant treatment in in vitro and ex vivo models, showing that anti-depressants may have therapeutic potential for treating diseases where activated microglia play a key role. However, it is crucial to note that microglia activation is heterogeneous in humans and that different subpopulations of microglia play different roles in both beneficial and harmful modes. Hence, further investigations into the underlying mechanisms are needed to see if anti-depressants could have benefits in neurodegenerative diseases [43,44]. 

Hypertension was detected as the highest scoring initial indication of drugs repurposed not only for AD but also for PD. There are many studies and meta-analyses available that show the association between antihypertensives and AD and specifically the effect that they have in decreasing the risk of AD [15,16,17]. Additionally, studies have shown that HD patients taking antihypertensives had decreased motor, cognitive and functional symptoms compared to HD patients who were untreated. In addition, these patients also presented with a later age of onset of the disease. A more thorough investigation is necessary to detect the underlying mechanisms of how antihypertensives can be used for the benefit of patients with different neurodegenerative diseases [45]. 

Moreover, topoisomerase inhibitor was detected as the highest-scoring MoA in both HD and MS. DNA topoisomerases are enzymes that control the topology of DNA in all cells and are essential for the smooth function of the cells. Due to this essentiality, topoisomerases have become key drug targets for many diseases, and these include several neurodegenerative diseases such as MS. The DNA topoisomerase inhibitor mitoxantrone is a drug used for patients with worsening relapsing–remitting and secondary progressive Multiple Sclerosis and hepatocellular carcinoma [21,22]. Additionally, it has been shown that the use of topotecan, another topoisomerase I inhibitor, improves motor and behavioural abnormalities of a transgenic HD mouse model, along with the gain of body weight and brain weight [46]. Another topoisomerase inhibitor, amsacrine, is used to treat acute leukaemia in adults. Acute lymphoblastic leukaemia (ALL) was one of the common initial indications detected between HD and AD. Since topoisomerase inhibitors are used for both neurodegenerative diseases and leukaemias, it could be indicative that there are opportunities to repurpose drugs between these diseases. 

Additionally, sodium channel blocker was detected as a highly scored MoA for MS and HD. Sodium channel dysfunction is very common in many neurodegenerative diseases such as AD, PD, ALS, MS and sodium channel blockers and could be potentially used for treating a wide range of neurodegenerative diseases [47]. Further studies on neuroprotection as a therapeutic intervention aimed at slowing or even halting the progression of neurodegeneration are needed in this direction.

Regarding pathways, amphetamine addiction was detected as highly scored for AD, PD and MS. Amphetamine exposure has been shown to have addictive effects and be associated with neuroinflammation, while studies have shown an increased risk of PD in amphetamine abusers [48]. Additionally, it has been shown that exposure to a more dangerous form of amphetamine causes Aβ42 formation, a key pathological hallmark in AD [49], and tau protein increase [50].

Lastly, glutamate release inhibitors were found in AD and PD, among others, whereas glutamate inhibitors were found in HD and MS. Glutamate is the predominant excitatory neurotransmitter, and in normal situations it is maintained at low levels since excessive glutamate receptor activation can lead to cell death. In the case where there is excessive activation of glutamate receptors present, known as excitotoxicity, it has been associated with a variety of neurodegenerative diseases such as MS, AD and others [51]. Interventions that target glutamate receptors would potentially prevent this excessive activation and, therefore, alleviate excitotoxicity, something that could be beneficial against neurodegenerative diseases in the future. 

Following the creation of the super-reference table, we detected groups of top drugs for each disease based on the final Total Composite Score. From this analysis, we detected common drugs at least in two diseases. For example, mianserin, with a score of 0.8 for AD and a score of 0.7 for PD, was detected. Mianserin is a tetracyclic anti-depressant, and it strongly stimulates the release of norepinephrine [52]. Furthermore, ketanserin, gr-113808, gr125487, idalopirdine, r-1485, rs-23597-190, rs-39604, sb-203186, sb-258585, sb-271046, sb-399885 and sb-742457 with a score of 0.7 for AD and PD, were detected. Ketanserin is a selective serotonin receptor antagonist. Idalopirdine is a potent and selective 5-HT_6_ receptor that, however, has been discontinued for AD [53]. The majority of these drugs are serotonin inhibitors or antagonists.

In addition, the group of the drugs iloperidone, pipotiazine–palmitate, clozapine, olanzapine, blonserin, paliperidone, lurasidone and others show high scores in AD, PD and HD with a score of 0.7 for AD and scores of 0.6 for both PD and HD. Iloperidone, clozapine, olanzapine and paliperidone are atypical antipsychotics used for the treatment of schizophrenia symptoms. Iloperidone was also detected by our previous computational drug repurposing study [42] as the second highest scoring candidate drug for Braak stage V–VI (severe) AD. Additionally, clozapine therapy appeared to be beneficial in treatment-resistant agitation in patients with dementia [54]. Pipotiazine–palmitate is used for the maintenance treatment of chronic non-agitated schizophrenic patients [55]. All of these drugs are used in the treatment of psychotic and schizophrenia symptoms.

Moreover, we compared the output of our methodology with existing tools, such as DrugShot [2] and DrugEnrichr [56]. When comparing our results with DrugShot, a web-based server application that allows the extraction of ranked lists of drugs based on the search of a biomedical term, such as the disease of interest, we find both common and complementary outcomes. The top 200 drugs for AD based on the Total Composite Score and the top 200 drugs based on the publication count extracted from DrugShot were compared, and we detected 18 common drugs between the two lists. Most of these drugs belong to the group of anti-depressive or antipsychotic drugs, such as mirtazapine, aripiprazole, quetiapine, olanzapine, risperidone and others. Moreover, drugs such as amitriptyline, which is used for pain syndromes such as fibromyalgia; lamotrigine, which is used for epilepsy and bipolar disorder; and celecoxib, a nonsteroidal anti-inflammatory drug, were detected as common between the two lists, among others.

In our current study, four neurodegenerative diseases were used as case studies. For AD and PD, more data were available, specifically for CDRS, whereas for HD and MS, the two more under-represented diseases, less data were available regarding the CDRS collection method. This makes the use of D^R^e^A^mocracy more informative for AD and PD since more lists of repurposed drugs were available. The more drug lists there are available, the more accurate the methodology. Additionally, D^R^e^A^mocracy leverages the existing knowledge to better understand where current research is currently focusing on in both CTS and CDRS studies. Therefore, these findings do not include all possible mechanisms behind neurodegeneration; yet, they provide an overview of what most of the studies support at the current time. 

Our method allows researchers to compare their drugs based on prior knowledge from studies. What we can see from our findings is that most of the time, there is an analogous trend in the frequency between the two. These results suggest that the CDRS and CTS are consistent but not redundant and, hence, complement each other. A combination of the two can enrich the individual information that each collection method can give and, therefore, can lead to a more complete result. Nevertheless, it is very important to note that the addition of any other collection method such as experimental in vitro drug screens could further enhance the output of D^R^e^A^mocracy.

The limitations of the present study include the restriction of using a small, although curated, library of 6798 molecules, known as the Drug Repurposing Hub Database. This means that drugs that need to be tested may not be included in the database, and, therefore, D^R^e^A^mocracy cannot score them. Moreover, drug names are used in their generic form, and, hence, a unique drug ID-based method, given that this is supported by the required databases, should be used in the future. However, this study offers the ability to access information on what has been discovered so far. Future research should focus on the generation of other reference tables by collecting information on other diseases and also using the final Total Composite Score in combination with other scores such as the scoring scheme proposed in our previous work [42]. In addition, further experimental testing is needed to validate the results of this study.

## 4. Materials and Methods

**Figure 7:**
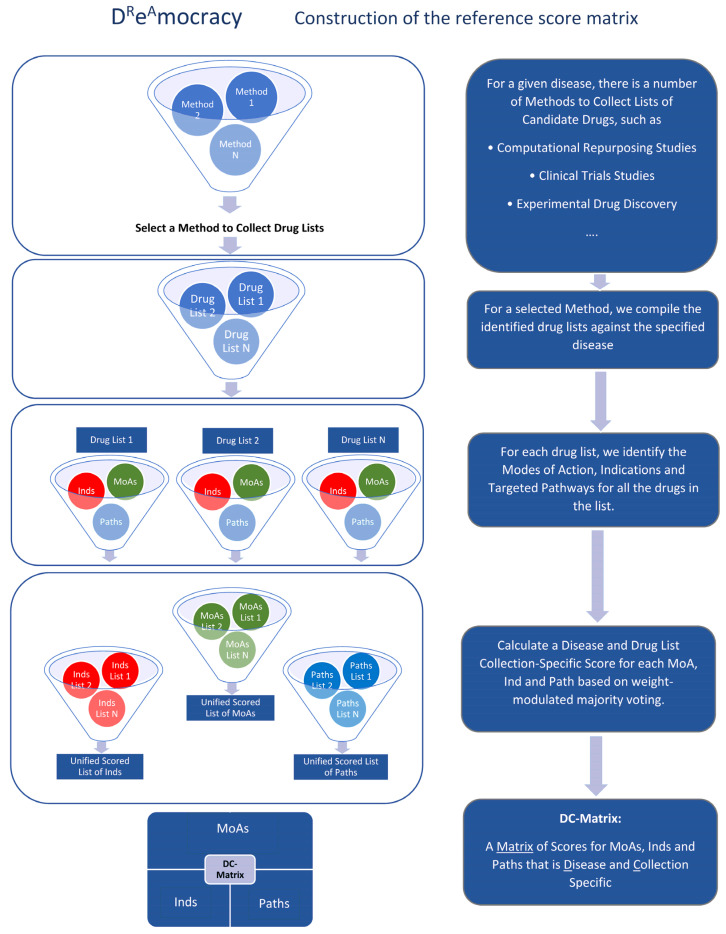
D^R^e^A^mocracy pipeline: Construction of the Reference Score Matrix. Initially, two different methods are applied to collect drug lists: computational drug repurposing studies lists (CDRS; Method 1) on AD, PD, HD and MS, as well as clinical trial studies (CTS; Method 2), for these diseases. Any method that generates drug lists can also be used (Method N). For each drug list produced via each method, we extracted the mechanisms of action (MoAs), initial indications (Inds) and targeted pathways (Paths) for each drug of the list. We then calculated a specific score for each MoA, Ind and Path (DC score) based on weight-modulated majority voting. Lastly, a DC-matrix that includes the DC scores is generated for each disease and each method.

D^R^e^A^mocracy integrates disease-specific drug lists generated by various drug discovery approaches. The drug collection methods that are selected in this work are (1) drug lists from the computational drug repurposing studies (CDRS) and (2) drug lists from the clinical trial studies (CTS). In addition, there is an option to generate a consensus scheme between the data from the two methods. The general pipeline of D^R^e^A^mocracy is presented in Figure 7. In Step 1, the construction of the reference score-matrix is carried out by choosing the method of interest to collect lists of drugs. These lists could be from CDRS, CTS, experimental drug discovery or any other method available that generates drug lists. The selected diseases for this purpose belong to the spectrum of neurodegeneration, as previously mentioned. For this purpose, we used CDRS and CTS for AD, PD, HD and MS. In Step 2, for each drug list for each method, we identified the MoAs, Inds and Targeted Paths for all the drugs in the list. In Step 3, we calculated a disease/collection-specific score for each MoA, Ind and Path, known as the DC score, based on weight-modulated majority voting. Lastly, in Step 4, a DC-matrix, which is a matrix of DC scores for MoAs, Inds and Paths that is disease and method-specific, was generated.

**Figure 8:**
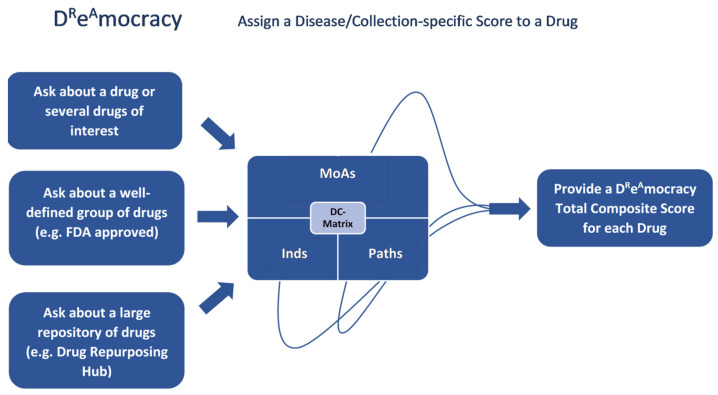
D^R^e^A^mocracy pipeline: Assigning a Disease/Collection-specific Score to a drug. We assign a disease/collection-specific score for each Drug based on the DC-matrices of D^R^e^A^mocracy. A Composite score is then generated to combine scores for MoAs, Paths and initial Inds for both CTS and CDRS lists. A final Total Composite Score for the Composite CTS and Composite CDRS score is generated.

The second part of the pipeline (Figure 8) included the assignment of a disease/collection-specific score to a drug, based on the DC-matrices of D^R^e^A^mocracy. For any drug or set of drugs of interest, one can query the D^R^e^A^mocracy-generated reference table to obtain the respective Total Composite Scores of the drugs based on the disease of interest.

### 4.1. Data Selection

Drug lists, both CDRS and CTS, contain generic drug names, obtained either from computational drug repurposing publications or clinical trials, respectively. Regarding the CDRS, proposed tables of repurposed drugs from applicable research papers were downloaded. Initial data were retrieved from PubMed (accessed on 8 January 2022) using the query type: (repurpos*[tiab] OR reposition*[tiab]) AND (the disease of interest e.g., Alzheimer*[tiab]). The output of the query was filtered to keep only research papers describing computational drug repurposing results for each of the selected diseases published in the past 10 years (Supplementary Tables S14–S17). To handle any inconsistencies, these drug lists were parsed against the Drug Repurposing Hub Database, and the SMILES format of the drugs was extracted, along with their generic drug name. Drugs were manually checked to see whether any drug synonyms were present. The same path was followed for the CTS. Clinical trial information was downloaded from www.clinicaltrials.gov as of 24th of January 2022 for each disease separately. The resulting text tables were downloaded and manually curated to keep only studies whose status was either “Completed”, “Actively Recruiting”, “Recruiting”, “Not yet recruiting” or “Active, not recruiting”. Studies that were “Suspended”, “Withdrawn” or “Terminated” were discarded. Moreover, in the interventions tab, only “drugs” were kept. Unique drugs were saved by keeping the clinical trial that was in the highest phase if multiple studies were available for the same drug. Information regarding the drug’s name, the status of the drug, and the phase in the clinical trial pipeline were kept. Only small molecules (“drugs”) were kept using the “interventions column”.

### 4.2. Construction of the Reference Score Matrix (DC-Matrix)

The drug collection for each disease (AD, PD, MS, HD) and each method (CDRS, CTS) was searched within the Drug Repurposing Hub Database (https://repo-hub.broadinstitute.org/repurposing, accessed on 2 February 2022), a comprehensive library with a total of 6798 drug annotations that are hand-curated, and their entities have been experimentally confirmed with the literature-reported targets. The types of data that were extracted from the drugs include structural information (SMILES), molecular mechanisms of action (MoAs), targets (and also pathways) and the initial indications that these drugs were used prior to drug repurposing. Drug indications, modes of action and targets were extracted for each drug in each list. Using the drug target information from the database, we mapped the pathways that the drug targets are involved in using EnrichR R package (15), and we collected all pathways of which the drug targets were members. Then, for each disease, a table of ranked lists for (a) Indications (Inds), (b) Mechanisms of Action (MoAs) and (c) Pathways (Paths) per disease were generated via (1) the CDRS method and (2) the CTS method. These reference tables, also known as DC-matrices (Drug List Collection Matrices), were prepared for each of the four neurodegenerative diseases. The frequency (Freq) of the drug information (Ind, MoA, Path) and the number of initial studies (ListCount) introducing this drug information were calculated. Both metrics, Freq and ListCount, were then normalised in the unit interval. The lists were ranked based on the normalised frequency (Norm_Freq) and the normalised count of lists (Norm_ListCount) according to the following equation (Equation (1)):

(1) $${DC\;Score}_{i}=W_{F}*{Norm_{Freq}}_{i}+W_{LC}*{Norm_{ListCount}}_{i},i\in \left\{moAs,inds,paths\right\}$$

where W_F_ and W_LC_ are the weights of importance of the normalised frequency and normalised count of lists for the MoAs, Inds and Paths. In this first application of the method, we set W_F_ = 0.4 and W_LC_ = 0.6. A higher weight was given to the ListCount because of the assumption that the detection of a signature in as many drug lists as possible is more important than the frequency of the signature for a single study/drug list.

Finally, to avoid random insertions in the DC-matrix lists, these scored lists were filtered using a hypergeometric test. Then, we kept only the MoAs, Inds and Paths with a hypergeometric test *p*-value lower than 0.05.

### 4.3. Assign a Disease/Collection-Specific Score to a Drug

Figure 2 describes the case where we can ask D^R^e^A^mocracy about (1) a drug or several drugs of interest, (2) a well-defined group of drugs (e.g., FDA approved) and (3) a large repository of drugs (e.g., the Drug Repurposing Hub). To proceed with the drug(s) scoring against a selected disease, we extract the corresponding Inds, MoAs and Paths of the drug(s) and we combine the corresponding MoAs, Paths and Inds scores from the DC-matrix into a Composite Score for each drug against the selected disease using the following equation (Equation (2)):

(2) $$Composite\;Score=W_{1}*ScoreOfPaths+W_{2}*ScoreOfMoAs+W_{3}*ScoreOfInds$$

where, in this first application of the method, we set W_1_ = 0.4, W_2_ = 0.4 and W_3_ = 0.2. Paths and MoAs were given equal and higher weights than the initial Inds since the first two give an actual description of the features of the drug rather than the initial use of the drug. The weights in Equation (2) are designed to be used as user-defined options to implement different strategies for scoring the findings from previous drug discovery efforts.

In the case of the combination of all the drug collection methods, a Total Composite Score can be calculated as an average of the Composite Scores for each data collection method (e.g., CDRS and CTS), as shown in the following equation (Equation (3)):

(3) $$Total\;Composite\;Score=\frac{1}{N}\cdot \sum_{i}{Composite\;Score}_{i}$$

i=1,…,N possible collection methods of drug lists.

In D^R^e^A^mocracy, N = 2 was used. However, this equation can be extended to cases where more methods might potentially be used.

### 4.4. Drug Repurposing Hub through the lens of D^R^e^A^mocracy

**Figure 9:**
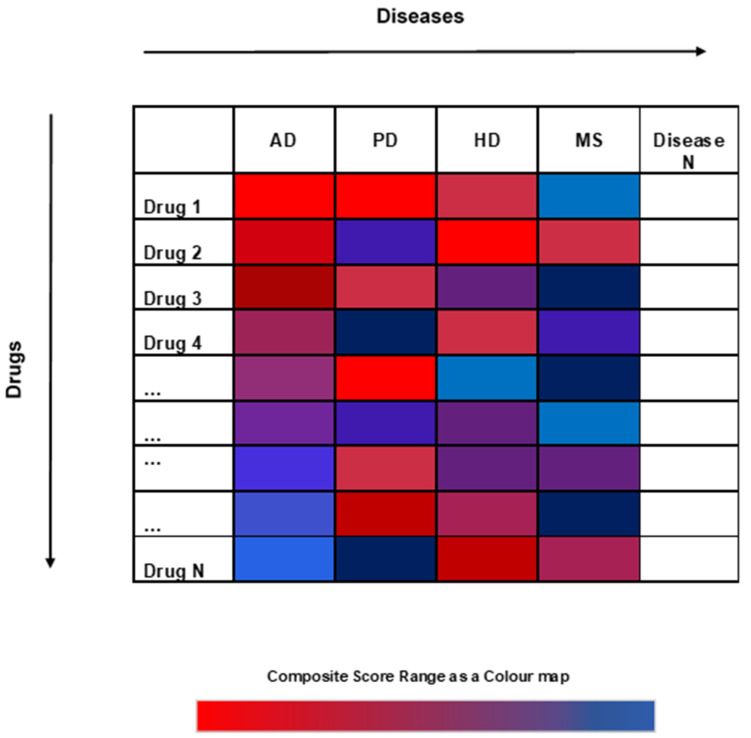
Schematic for a large-scale application of D^R^e^A^mocracy for various diseases. By following the D^R^e^A^mocracy pipeline, the Total Composite Scores can be calculated for any disease of interest that has available prior knowledge, either on clinical trial studies or on computational drug repurposing studies. The Drug Repurposing Hub was used in this work to extract the available information regarding the drugs. In the current scheme, the drugs in the table are considered sorted based on their score in the first column (AD).

We performed a large-scale computation by calculating the Total Composite Score for each drug in the Drug Repurposing Hub with a total of 4421 drugs for four diseases: AD, PD, HD and MS. The whole pipeline of D^R^e^A^mocracy, however, can be applied in multiple diseases given the sufficient availability of drug-related a priori information. This can be extended to a greater number of diseases, as shown in Figure 9 (N diseases), and any other database with the same type of data can be used. Moreover, in addition to the different diseases that can be tested, different methods to collect drug lists can be used, such as experimental drug discovery studies. Notably, a better Composite Score can be given when more data are available for the disease of interest, as a higher diversity can be added to the methodology. Therefore, to obtain more reliable results, it is necessary to create a minimum of three drug lists per disease to proceed with the creation of its reference table.

In case the user wants to use our approach while selecting different weights, a simple R shiny web application is available. The user can choose a drug of interest for one of the four diseases available, the weights of MoAs, Inds and Paths and the type of studies (CDRS and/or CTS) using the GitHub code (see data and code availability).

## 5. Conclusions

Overall, our study presents a novel methodology that takes advantage of the aggregated information from the drug repurposing lists already available, as well as from clinical trial studies, towards the generation of a dynamic reference matrix with enhanced resolution regarding the disease-related frequencies of drug characteristics. We expect that our proposed methodology will be applied to many diseases of interest in the future and will serve as a reference for those interested in testing their drugs against the drug-related information available on these diseases. We would like to state that this is a computational methodology that highlight good candidates as possible repurposed drugs. Nevertheless, further experimental testing is necessary to end up with lists of actual repurposed drugs. 

## Figures and Tables

**Table 1:** Super-reference table. A snapshot of the super-reference table (top 20 drugs ranked by each disease’s Total Composite Score separately). In this sub-table (See Supplementary Table S13), the final Total Composite Score is shown for all four neurodegenerative diseases, using the Drug Repurposing Hub as the input to D^R^e^A^mocracy.

Drug Name	AD Total Composite Score	Drug Name	PD Total Composite Score	Drug Name	HD Total Composite Score	Drug Name	MS Total Composite Score
agomelatine	0.85	apomorphine	0.73	chlorpromazine	0.68	zonisamide	0.55
mirtazapine	0.85	pramipexole	0.73	fluphenazine	0.68	disopyramide	0.52
vortioxetine	0.83	lisuride	0.72	perphenazine	0.68	priralfimide	0.52
aripiprazole	0.83	terguride	0.70	trifluoperazine	0.68	dalfampridine	0.51
mianserin	0.80	bromocriptine	0.70	risperidone	0.63	chloroprocaine	0.49
sarpogrelate	0.77	ropinirole	0.68	pimozide	0.63	valproic-acid	0.48
cyamemazine	0.76	rotigotine	0.68	chlorprothixene	0.58	cinchocaine	0.46
clozapine	0.74	piribedil	0.68	clozapine	0.58	ibutilide	0.45
loxapine	0.73	fenoldopam	0.67	flupentixol	0.58	haloperidol–decanoate	0.45
ketanserin	0.73	mirtazapine	0.66	iloperidone	0.58	troglitazone	0.44
methysergide	0.72	a-412997	0.64	levomepromazine	0.58	spironolactone	0.42
quetiapine	0.72	abt-724	0.64	olanzapine	0.58	tesaglitazar	0.42
ziprasidone	0.72	etilevodopa	0.64	pipotiazine	0.58	rosiglitazone	0.42
blonserin	0.72	ro-10-5824	0.64	pipotiazine–palmitate	0.58	pioglitazone	0.42
paliperidone	0.72	metixene	0.64	promazine	0.58	flibanserin	0.41
gr-113808	0.72	agomelatine	0.64	spiperone	0.58	phenacemide	0.40
gr125487	0.72	mesulergine	0.63	thioproperazine	0.58	eslicarbazepine–acetate	0.40
idalopirdine	0.72	talipexole	0.62	thiothixene	0.58	oxcarbazepine	0.40
r-1485	0.72	dr-4485	0.62	zuclopenthixol	0.58	procaine	0.40
rs-23597-190	0.72	sb-269970	0.62	amisulpride	0.57	amiloride	0.39

## Data Availability

https://github.com/kyriaki94/DReAmocracy, accessed on 29 April 2024.

## Supplementary Materials

The following supporting information can be downloaded at: https://www.mdpi.com/article/10.3390/ijms25105319/s1.

## Abbreviations

AD	Alzheimer’s Disease
ALL	Acute lymphoblastic leukaemia
ALS	Amyotrophic Lateral Sclerosis
AMPK	AMP-activated Protein Kinase
CDRS	Computational drug repurposing studies
CMap	Connectivity Map
CTCL	T-cell lymphoma
CTS	Clinical trial studies
DC Score	Drug/Collection-specific Score
EIB	Exercise-induced bronchoconstriction
FDA	Food and Drug Administration
HD	Huntington’s Disease
HMGCR	3-hydroxy-3-methylglutaryl-CoA reductase
Ind	Initial indication
MoA	Mechanism of action
MS	Multiple Sclerosis
Path	Pathway
PD	Parkinson’s Disease
PPAR	Peroxisome Proliferator-activated Receptors
VMAT2	Vesicular monoamine transporter 2
