# Supplementary material for: DReAmocracy: A Method to Capitalise on Prior Drug Discovery Efforts to Highlight Candidate Drugs for Repurposing
Source: Int J Mol Sci. 2024 May 13;25(10):5319. doi: 10.3390/ijms25105319 (PMC11121186; doi:10.3390/ijms25105319)
Supplement: Supplementary file 1 [file ijms-25-05319-s001.zip › Supplementary_Materials.pdf]

Supplementary Materials for

D^Re^Amocracy: A Method to Capitalize on Prior Drug Discovery Efforts to Highlight Candidate Drugs for Repurposing

Frequency ranking comparison

Frequency comparison was performed only for AD and PD. For this comparison, only statistically significant features of drugs were used (hypergeometric test and a p-value <0.05). For AD Path comparison, we found a significant relationship using regression analysis (p-val= 4.751e-07) between frequencies of CDRS and CTS lists, with a r^2 of 0.9638± 0.071. R squared (r2) is a statistical test that shows how well the data fit the regression model (the goodness of fit).

For instance, frequency comparison, *serotonergic synapse, dopaminergic synapse* and *AMPK signalling pathway* are the top-scored Paths detected that have analogous frequencies in both CDRS and CTS. For AD initial Inds, we found a significant relationship (p-val= 4.535e-08) between frequencies of CDRS and CTS lists, with a r^2 of 0.9947±0.017. For instance, *depression* is the highest-scored pathway, with a normalised frequency score 0.85 in CTS and 0.45 in CDRS. In the CDRS list, *depression* scored third, however, it is the top-scored common MoA with CTS. Moreover, for the comparison of AD MoAs, we found a significant relationship (p-val= 0.008) between frequencies of CDRS and CTS lists, with a r^2 of 0.8533±0.18. For instance, *serotonin receptor antagonist* was found to be the top-scored MoA, with an analogous frequency in both CDRS and CTS (0.92 and 1 respectively). For comparison of the PD Paths, we found a significant relationship (p-val= 2.701e-06) between frequencies of CDRS and CTS lists, with an r^2 of 0.7146± 0.13. For instance, the *calcium signalling pathway* and *neuroactive ligand-receptor interaction* were detected as the top-scored Paths, with the former having a normalised frequency of 1 and 0.81 for CDRS and CTS respectively, and the latter having a normalised frequency of 1 for both CDRS and CTS. For PD initial Inds, *Alzheimer's disease* scored low in both CDRS and CTS (0.2 and 0.3 respectively). Lastly, for PD MoAs, we found a significant

relationship (p-val= 0.01593) between frequencies of CDRS and CTS lists, with a r^2 of 0.8904± 0.10. For example, *Dopamine receptor agonist* had a top score for CTS (score 1) whereas CDRS had a score of 0.47. Moreover, *serotonin receptor antagonist* had a score of 0.57 for CDRS and 0.75 for CTS. These results show that the common signatures between CDRS and CTS lists have a consistent ranking in both lists.

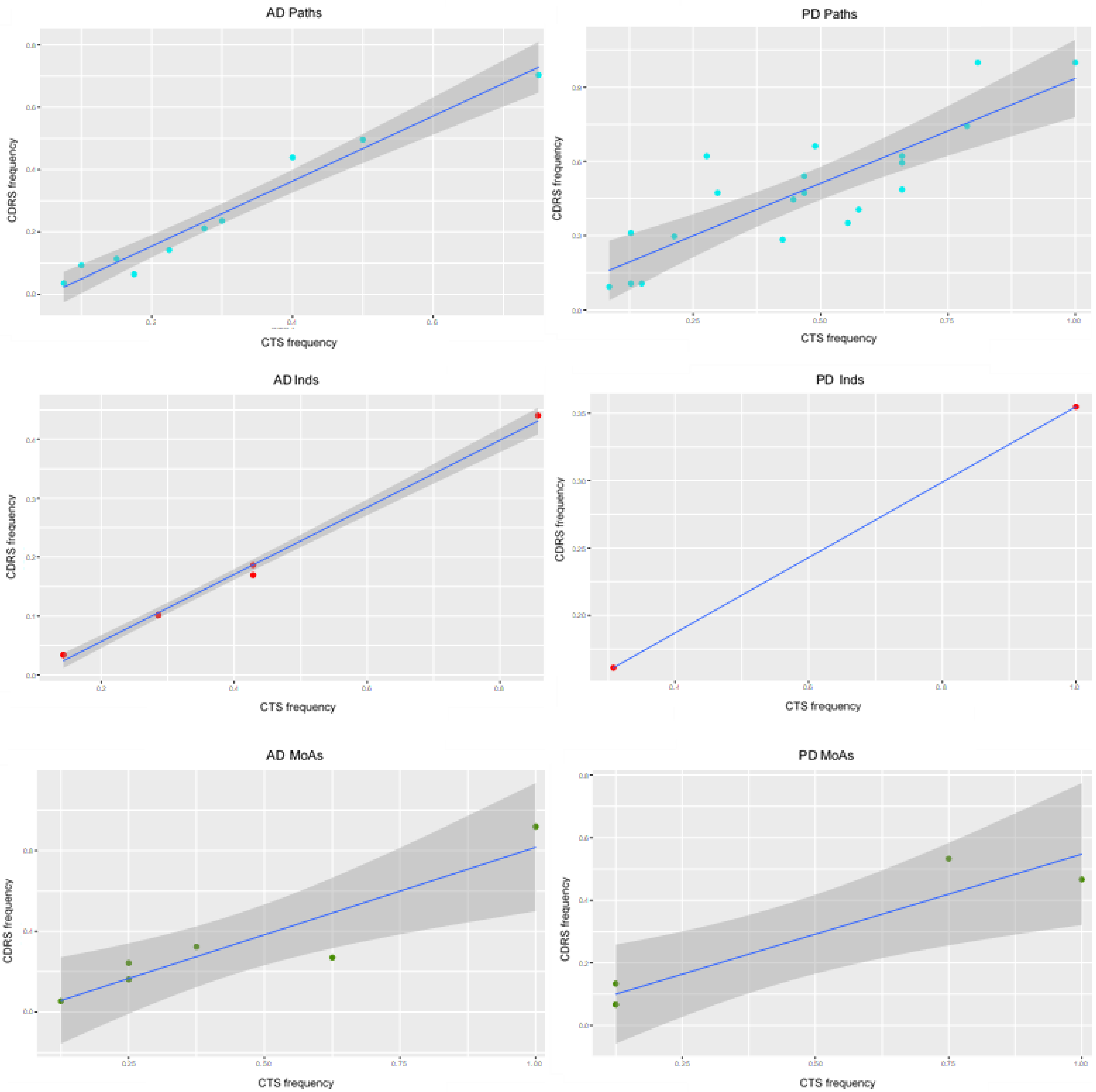

Figure S1: Frequency comparison in AD and PD. Points represent the normalised frequency of signatures in CDRS (y-axis) and CTS (x-axis). Linear regression is used to model the relationship between the two variables and estimate the value of a response by using the best fitting line. Blue colour dots depict Paths, red colour dots Inds and green colour dots MoAs.
